# Epidemiology and outcomes of culture-positive bloodstream pathogens prior to and during the SARS-CoV-2 pandemic: a multicenter evaluation

**DOI:** 10.1186/s12879-022-07810-8

**Published:** 2022-11-11

**Authors:** Karri A. Bauer, Laura A. Puzniak, Kalvin C. Yu, Lyn Finelli, Pamela Moise, ChinEn Ai, Janet A. Watts, Vikas Gupta

**Affiliations:** 1 Merck & Co, Inc, Kenilworth, NJ USA; 2 Becton, Dickinson and Company, 1 Becton Drive, Franklin Lakes, NJ 07417 USA

**Keywords:** Blood stream infection (BSI), Hospital onset BSI, Community-onset BSI, COVID-19 pandemic, Bacterial pathogens, Fungal pathogens, Patient outcomes

## Abstract

**Background:**

Bloodstream infections (BSIs) are an important cause of morbidity and mortality in hospitalized patients. We evaluate incidence of community- and hospital-onset BSI rates and outcomes before and during the SARS-CoV-2 pandemic.

**Methods:**

We conducted a retrospective cohort study evaluating patients who were hospitalized for ≥ 1 day with discharge or death between June 1, 2019, and September 4, 2021, across 271 US health care facilities. Community- and hospital-onset BSI and related outcomes before and during the SARS-CoV-2 pandemic, including intensive care admission rates, and overall and ICU-specific length of stay (LOS) was evaluated. Bivariate correlations were calculated between the pre-pandemic and pandemic periods overall and by SARS-CoV-2 testing status.

**Results:**

Of 5,239,692 patient admissions, there were 20,113 community-onset BSIs before the pandemic (11.2/1000 admissions) and 39,740 (11.5/1000 admissions) during the pandemic (*P* ≤ 0.0062). Corresponding rates of hospital-onset BSI were 2,771 (1.6/1000 admissions) and 6,864 (2.0/1000 admissions; *P* < 0.0062). Compared to the pre-pandemic period, rates of community-onset BSI were higher in patients who tested negative for SARS-CoV-2 (15.8/1000 admissions), compared with 9.6/1000 BSI admissions among SARS-CoV-2-positive patients. Compared with patients in the pre-pandemic period, SARS-CoV-2-positive patients with community-onset BSI experienced greater ICU admission rates (36.6% vs 32.8%; *P* < 0.01), greater ventilator use (10.7% vs 4.7%; *P* < 0.001), and longer LOS (12.2 d vs 9.1 d; *P* < 0.001). Rates of hospital-onset BSI were higher in the pandemic vs the pre-pandemic period (2.0 vs 1.5/1000; *P* < 0.001), with rates as high a 7.3/1000 admissions among SARS-CoV-2-positive patients. Compared to the pre-pandemic period, SARS-CoV-2-positive patients with hospital-onset BSI had higher rates of ICU admission (72.9% vs 55.4%; *P* < 0.001), LOS (34.8 d vs 25.5 d; *P* < 0.001), and ventilator use (52.9% vs 21.5%; *P* < 0.001). *Enterococcus* species, *Staphylococcus aureus, Klebsiella pneumoniae,* and *Candida albicans* were more frequently detected in the pandemic period.

**Conclusions and relevance:**

This nationally representative study found an increased risk of both community-onset and hospital-onset BSI during the SARS-CoV-2 pandemic period, with the largest increased risk in hospital-onset BSI among SARS-CoV-2-positive patients. SARS-CoV-2 positivity was associated with worse outcomes.

**Supplementary Information:**

The online version contains supplementary material available at 10.1186/s12879-022-07810-8.

## Introduction

Bloodstream infections (BSIs) are a major cause of morbidity and mortality in the United States [1, 2]. Prior to the COVID-19 pandemic, BSIs and septicemia affected 1.5 million Americans annually [3, 4]. Recent studies have reported BSI rates among hospitalized patients with COVID-19 of 7 to 15%, and estimates suggest that up to 50% of patients who die from COVID-19 have a coinfection with an additional pathogen [5, 6].

Data also indicate that patients with COVID-19 and BSI have worse outcomes [6–8]. For example, a multicenter, retrospective study reported that adult patients who were hospitalized with severe COVID-19 and secondary BSIs presented with more severe illness and had longer hospital stays and higher mortality rates [7]. Similarly, a study found that SARS-CoV-2-positive patients early in the pandemic had higher rates of hospital-onset infections, greater antimicrobial usage, and extended hospital and intensive-care unit length of stay (LOS), compared with SARS-CoV-2-negative or untested patients [8]. Another study reported a 3.9-fold increase in BSI among hospitalized patients who tested positive for SARS-CoV-2 compared to those who tested negative [6].

However, most studies on the epidemiology of BSI during the COVID-19 pandemic have been of short duration and report on the early period of the pandemic [7–9], highlighting a need for data that capture the impact of clinically relevant COVID-19 variants, including the Delta variant, and reflect the use of immunomodulatory agents, which could affect the risk of secondary infections [10].

To address these limitations, we evaluated the epidemiology and outcomes of culture-positive gram-negative, gram-positive, and fungal/yeast pathogens from a blood source in patients hospitalized prior to and during the SARS-CoV-2 pandemic across 271 facilities in the United States.

## Methods

### Study design

This multicenter, retrospective cohort analysis included all hospitalized adults aged ≥ 18 years from 271 US facilities included in the BD Insights Research Database (Becton, Dickinson and Company, Franklin Lakes, NJ), which includes both small and large medical care facilities in rural and urban areas throughout the United States (Additional file 1: Table S1). This electronic surveillance system and clinical research database, which has been previously described [8, 11, 12], is exempted from patient consent requirements because it uses deidentified data. This retrospective, deidentified data set was approved, and informed consent requirements were waived by the New England Institutional Review Board (Wellesley, MA, USA; IRB No. 120180023). We followed the Strengthening the Reporting of Observational Studies in Epidemiology (STROBE) guidelines for cohort studies to develop this report [13].

Eligible admissions included subjects with ≥ 1 inpatient stay with a record of discharge or death between July 1, 2019, and September 4, 2021. The pre-pandemic period was defined as July 1, 2019, to February 29, 2020, and the pandemic period was defined as March 1, 2020, to September 4, 2021. All admissions with an incident BSI infection, defined as a non-contaminated first positive blood culture for gram-negative or gram-positive bacterial pathogens, fungal pathogens, or yeast pathogens, were included in the analysis. Microbiology results likely associated with a blood contaminant were excluded by a previously described methodology that uses time of collection, source, microorganism type, and numbers of microorganisms in a culture to flag likely contaminated samples [14]. Monomicrobial BSI with the gram-negative pathogens, *Enterobacterales* (*Citrobacter freundii, Escherichia coli, Enterobacter cloacae, Klebsiella pneumoniae, Klebsiella oxytoca, Klebsiella aerogenes, Morganella morganii, Proteus mirabilis, Providencia stuartii, Serratia marcescens*), *Acinetobacter baumannii* species*, Bacteroides fragilis, Pseudomonas aeruginosa*, and *Stenotrophomonas maltophilia*; the monomicrobial gram-positive pathogens *Enterococcus* spp, group A *Streptococcus*, group B *Streptococcus*, *Staphylococcus aureus*, and *Streptococcus pneumoniae*; the monomicrobial fungus or yeast pathogens, non-*Candida albicans*, *Candida albicans*, and other *Candida* were included in the analysis. Polymicrobial results were included cases in which > 1 designated pathogen was obtained from first positive blood culture during the admission. For the purposes of comparison, patients were categorized into 3 groups: (1) SARS-CoV-2 positive, (2) tested and SARS-CoV-2 negative, and (3) SARS-CoV-2 untested. SARS-CoV-2 infection was defined as a positive SARS-CoV-2 antigen or polymerase chain reaction (PCR) test 7 days prior to admission or during hospitalization.

BSIs were defined as community-onset infections (COBSI) if the blood culture was collected ≤ 2 days from admission and as hospital-onset infections (HOBSI) if the blood culture was collected > 2 days from admission. All results from microbiology testing were obtained from analyses performed by local microbiology labs in the hospitals included in the BD Insights Research Database.

### Outcomes

The primary outcome was to compare the overall rate of BSI during the pre-pandemic period with the rate of BSI in SARS-CoV-2-positive, SARS-CoV-2-negative, and SARS-CoV-2-untested patients during the pandemic period. Secondary outcomes included the rates of COBSI and HOBSI, the time to collection of HOBSIs, and the characteristics and outcomes of patients with COBSI and HOBSI.

In-hospital mortality was reported in cases with a designation of mortality, presence in the morgue, or death in Admission, Discharge, and Transfer (ADT) data feeds [11]. We evaluated in-hospital mortality (available for patients tested for SARS-CoV-2), hospital LOS, and intensive care unit (ICU) LOS in all patients and stratified by SARS-CoV-2 testing status during the pandemic period. LOS was calculated using hospital ADT data as the difference between admission and discharge dates.

Maximum laboratory values assessed within the first 3 days of admission, except for diabetes, were used as surrogates for comorbidities, defined as severe underlying illnesses or conditions, during the admission period (Additional file 2: Table S2) [11]. We evaluated the impact renal insufficiency or failure, diabetes, sepsis, liver dysfunction, myocardial inflammation or suspected heart failure, and cytokine stimulation (Additional file 2: Table S2).

Based on previously published criteria and a lack of timely data within the database to identify ventilator use, ventilator use was assumed in patients who were (a) started on intravenous (IV) push sedation medications (propofol, lorazepam, midazolam, ketamine, or dexmedetomidine,) or IV opioids (fentanyl, sufentanil, remifentanil, or hydromorphone) with a duration of at least 24 h, and (b) who had at least 2 arterial blood gas results collected at least 24 h apart, the first blood gas collected on the first day of sedation medication [11].

### Statistical analysis

Bivariate correlations were calculated between the pre-pandemic and pandemic periods overall and by SARS-CoV-2 testing status for age, gender, prior admission, underlying conditions, LOS, ventilation status, and ICU status. Chi square or Fisher’s exact tests were used to perform bivariate comparisons between categorical subgroups, while continuous variables were analyzed using Kruskal–Wallis tests and Wilcoxon tests were used for pairwise comparisons. Two sample t-tests were used to compare the means of two samples. All statistical tests were conducted using a pre-specified two-tailed alpha level of 0.05. Statistical analyses were performed using R (R Ver. 4.1.2, R Foundation for Statistical Computing, Vienna, Austria), with RStudio (Boston, MA).

## Results

There were 1,789,449 patient admissions during the pre-pandemic period (July 1, 2019, to February 29, 2020) and 3,450,243 admissions during the pandemic period (March 1, 2020, to September 4, 2021). Most (65%) hospitals included in the analysis were urban and 75% had 300 or fewer beds **(**Additional file 1: Table S1).

BSIs were detected in 22,884 patient admissions (12.79/1000 admissions) in the pre-pandemic period and in 46,568 admissions (13.50/1000 admissions) during the pandemic period (*P* ≤ 0.0001). Both COBSI and HOBSI were observed more frequently during the pandemic than the pre-pandemic period (COBSI: 11.51 vs 11.24 per 1000 admissions, *P* ≤ 0.0022; HOBSI: 1.99 vs 1.55 per 1000 admissions; *P* < 0.0001 for both comparisons), as shown in Table 1.

**Table 1:** Patient admission rates with community-onset and hospital-onset BSI during the pre- and SARS-CoV-2 pandemic periods

Category	Pre-pandemic rate/1000 admissions (July 2019–February 2020) (n = 1,789,449 admissions)	SARS-CoV-2 pandemic rate/1000 admissions (March 2020-September 4, 2021) (n = 3,450,243 admissions)
Community-onset	11.24 (20,113)	11.51 (39,740)^a^
Hospital-onset	1.55 (2771)	1.99 (6864)^b^
Total	12.79 (22,884)	13.50 (46,568)^b^

^a^*P* ≤ 0.0022 and ^b^*P* < 0.0001 for post-SARS-CoV-2 period vs pre-SARS-CoV-2 period

### Epidemiology and pathogen distribution of COBSI before and during the SARS-CoV-2 pandemic

Mean ages of patients admitted for COBSI were comparable between the pre-pandemic and pandemic periods (65.1 vs 65.2 years; Table 2). Patients with COBSI during the pandemic period were more likely to be male (53.3% vs 52.0%; *P* < 0.05), have prior admissions in the previous 90 days (27.0% vs 22.7%; *P* < 0.001), and have ≥ 1 underlying condition (89.1% vs 86.3%; *P* < 0.001; Table 2).

**Table 2:** Epidemiology and outcomes of COBSI before and during SARS-CoV-2 periods by SARS-CoV-2 testing status

	July 2019-February 2020	March 2020–September 4, 2021			
	Pre-pandemic period	Pandemic period			
Characteristics		SARS-CoV-2 +	SARS-CoV-2 −	SARS-CoV-2 not tested	Total
	20,113	1638	27,476	10,590	39,704
Unadjusted BSI rate per 1000 admissions	**11.240**	**9.613**^**c**^	**15.818**^**c**^	**6.864**^**c**^	**11.508**^**a**^
Mean age ± SD, (IQR; median)	65.1 ± 16.7 (55–78; 67)	66.2 ± 16.2 (56–79; 68)^a^	65.2 ± 16.3 (55–77; 67)	64.6 ± 16.6 (55–77; 67)^a^	65.2 ± 16.4 (55–77; 67)
Males, n (%)	10,467 (52.0)	843 (51.5)	14,751 (53.7)^b^	5560 (52.5)	21,154 (53.3%)^a^
Prior 30-day admission, (%)	13.2% (2655)	16.7% (274)^c^	14.4% (3945)^c^	14.9% (1581)^c^	14.6% (5800)^c^
Prior 90-day admission (%)	22.7% (4572)	27.2% (445)^c^	26.9% (7393)^c^	27.3% (2889)^c^	27.0% (10,727)^c^
≥1 Underlying conditions^d^ (%)	86.3% (17,356)	94.6% (1550)^c^	90.7% (24,932)^c^	83.9% (8889)^c^	89.1% (35,371)^c^
LOS (days): avg ± SD (IQR; median)	9.1 ± 8.3 (4–11; 7)	12.2 ± 11.8 (5–15; 9)^c^	9.2 ± 8.6 (4–11; 7)	8.3 ± 7.8 (4–10; 6)^c^	9.6 ± 8.6 (4–11; 7)
% Ventilated^e^ (%)	4.7% (954)	10.7% (175)^c^	5.4% (1490)^c^	4.0% (424)^a^	5.3% (2089)^b^
ICU admission (%)	32.8% (6591)	36.6% (600)^b^	29.6% (8138)^c^	26.5% (2838)^c^	29.2% (11,576)^c^
Overall LOS (days): avg ± SD (IQR; median)	12.1 ± 10.6 (6–15; 9)	15.5 ± 14.9 (6–20; 12)^c^	12.5 ± 11.7 (6–15, 9)	11.3 ± 10.7 (5–14; 8)^c^	12.3 ± 11.7 (5–15; 9)
ICU LOS (days): avg ± SD (IQR; median)	4.7 ± 5.3 (2–6; 3)	6.5 ± 7.4 (2–8; 4)^c^	5.0 ± 6.8 (2–6; 3)^a^	4.6 ± 5.5 (1–6; 3)^c^	5 ± 6.6 (2–6; 3)^b^
Hospital mortality (%)	NA	20.7% (237/1,146)	7.0% (1324/18,843)	NA	7.8% (1567/20,064)

^a^*P* < .05; ^b^*P* < .01; ^c^*P* < .001 bivariate correlations compared to the pre-SARS-CoV-2 period

^d^Includes the following baseline conditions occurring within the first 3 days of hospital admissions as determined by the maximum value of surrogate laboratory results: renal insufficiency (serum creatinine [SCr] > 2.0 mg/dL [to convert to micromoles per liter, multiply by 88.4]); kidney failure (blood urea nitrogen > 100 and SCr > 3.0 mg/dL); suspected sepsis (lactic acid > 2.0 or > 4.0 mmol/L); suspected heart failure (brain-type natriuretic peptide [BNP] > 400 pg/mL [to convert to nanograms per liter, multiply by 1.0] or N-terminal pro BNP > 900 pg/mL); myocardial inflammation (doubling of troponin drawn within 6 h final value: troponin 0.4 ng/L [to convert to micrograms per liter, multiply by 1.0], troponin T15 ng/L [to convert to micrograms per liter, multiply by 1.0] in male patients, troponin T10 ng/L in female patients, troponin I > 0.04 ng/mL [to convert to micrograms per liter, multiply by 1.0]); liver dysfunction (any of the following: alanine aminotransferase > 60 U/L [to convert to microkatals per liter, multiply by 0.0167], aspartate aminotransferase > 80 U/L [to convert to microkatals per liter, multiply by 0.0167], serum albumin < 3.0 g/dL [to convert to grams per liter, multiply by 10], international normalized ratio > 2.0 [and not currently receiving warfarin, rivaroxaban, apixaban, edoxaban, or betrixaban]); cytokine stimulation (any of the following: fibrinogen < 250 mg/dL [to convert to grams per liter, multiply by 0.01], C-reactive protein > 7.0 mg/dL [to convert to milligrams per liter, multiply by 10], D-dimer [dimerized plasmin fragment D] > 1000 ng/mL [to convert to nanomoles per liter, multiply by 5.476], erythrocyte sedimentation rate > 30 mm/h, or triglycerides > 265 mg/dL [to convert to millimoles per liter, multiply by 0.0113])

^e^The patient was (a) started on intravenous (IV)/IV push (IVP) sedation medications (propofol, lorazepam, midazolam, dexmedetomidine, or ketamine) or IV/IVP opioids (fentanyl, remifentanil, sufentanil, or hydromorphone) with a duration ≥ 24 h and (b) ≥ 2 arterial blood gas results collected ≥ 24 h apart (on the first day of sedation medication and a subsequent result 24 h later)

There were 39,704 patient admissions with COBSI during the pandemic period. Rates of COBSI were highest among SARS-CoV-2-negative patients (15.8/1000 admissions), followed by SARS-CoV-2-positive patients (9.6/1000 admissions), and those not tested (6.86/1000 admissions).

SARS-CoV-2-positive patients were older (66.2 vs 65.1; *P* < 0.05) and were more likely to have had ≥ 1 underlying conditions (94.6% vs 86.3%; *P* < 0.001). During the pandemic period compared to the pre-pandemic period, COBSI rates were significantly higher among SARS-CoV-2-negative patients (15.8 vs 11.2; *P* < 0.001).

Significantly higher rates of COBSI admissions were seen during the pandemic period compared to the pre-pandemic period for the following pathogens (per 1000 admissions): *S aureus* (3.05 vs. 2.81), *K pneumoniae* (1.00 v. 0.93), *Enterococcus* spp (0.74 vs. 0.64), group B Strep (0.45 vs. 0.42), and polymicrobial pathogens (0.42 vs. 0.36), all *P* ≤ 0.0404, as shown in Table 3.

**Table 3:** Community-onset and hospital-onset BSI pathogen comparison pre-pandemic and pandemic periods

Pathogen	Pre-pandemic: July 2019-February 2020 (n = 1,789,449 admissions)		Pandemic period March 2020-September 4, 2021 (n = 3,450,243 admissions)	
	Community-onset BSI rate/1000 Adm (n)	Hospital-onset BSI rate/1000 Adm (n)	Community-onset BSI rate/1000 Adm (n)	Hospital-onset BSI rate/1000 Adm (n)
Gram-negative				
*E coli*	3.78 (6764)	0.23 410)	3.77 (13,001)	0.22 (766)
*K pneumoniae*	0.93 (1,662)	0.14 (250)	1.00 (3456)^a^	0.17 (597)^b^
*P aeruginosa*	0.39 (697)	0.08 (141)	0.37 (1275)	0.11 (384)^b^
*P mirabilis*	0.35 (622)	0.02 (39)	0.38 (1,294)	0.03 (89)
*E cloacae*	0.18 (318)	0.06 (102)	0.18 (610)	0.06 (207)
*S marcescens*	0.12 (208)	0.04 (64)	0.14 (477)^a^	0.06 (192)^b^
*B fragilis*	0.10 (171)	0.02 (35)	0.10 (339)	0.02 (65)
*K oxytoca*	0.09 (153)	0.02 (40)	0.11 (384)^a^	0.02 (65)
*K (E) aerogenes*	0.07 (126)	0.02 (41)	0.07 (236)	0.03 (88)
*A baumannii spp*	0.05 (91)	0.01 (22)	0.04 (141)	0.02 (56)
*P stuartii*	0.02 (33)	< 0.01 (3)	0.02 (86)	< 0.01 (14)
*S maltophilia*	0.02 (36)	0.01 (23)	0.02 (62)	0.01 (42)
*M morganii*	0.04 (70)	0.01 (10)	0.06 (191)^a^	0.01 (25)
*C freundii*	0.03 (49)	< 0.01 (8)	0.03 (103)	< 0.01 (16)
Gram-positive				
*S aureus*	2.81 (5035)	0.45 (812)	3.05 (10,511)^a^	0.58 (2,016)^b^
*Enterococcus spp*	0.64 (1152)	0.16 (283)	0.74 (2559)^a^	0.25 (874)^b^
*S pneumoniae*	0.42 (751)	0.01 (12)	0.23 (807)^a^	0.01 (39)
*Grp B Strep*	0.42 (759)	0.01 (26)	0.45 (1543)^a^	0.02 (59)
*Grp A Strep*	0.25 (443)	0.01 (12)	0.19 (651)	< 0.01 (13)
Fungus/Yeast				
Non-*C albicans*	0.11 (202)	0.10 (181)	0.10 (329)	0.15 (533)^b^
*C albicans*	0.07 (121)	0.08 (147)	0.05 (189)	0.13 (446)^b^
*Other—Candida*	< 0.01 (2)	< 0.01 (6)	< 0.01 (2)	< 0.01 (6)^b^

Pathogen	Pre-pandemic: July 2019- February 2020 (n = 1,789,449 admissions)		Pandemic period March 2020- September 4, 2021 (n = 3,450,243 admissions)	
	Community-onset BSI rate/1000 Adm (n)	Hospital-onset BSI rate/1000 Adm (n)	Community-onset BSI rate/1000 Adm (n)	Hospital-onset BSI rate/1000 Adm (n)
Polymicrobial	0.36 (648)	0.06 (104)	0.42 (1,458)^a^	0.08 (272)^b^

^a^*P* ≤ 0.0404 for community (CO) pandemic period vs. pre-pandemic period; ^b^*P* ≤ 0.0093 for HO pandemic period vs pre-pandemic period

### Epidemiology and pathogen distribution of HOBSI before and during the SARS-CoV-2 pandemic

There were 6,864 patient admissions with HOBSI during the pandemic period. Rates of HOBSI were highest among SARS-CoV-2-positive patients (7.3/1000 admissions), followed by SARS-CoV-2-negative patients (2.3/1000 admissions), and then those not tested (1.0/1000 admissions; Table 4).

**Table 4:** Epidemiology and outcomes of HOBSI before and during SARS-CoV-2 periods by SARS-CoV-2 testing status

Characteristics	July 2019-February 2020	March 2020-September 4, 2021			
	Pre-SARS-CoV-2	SARS-CoV-2 +	SARS-CoV-2-	SARS-CoV-2 not tested	Total
	2771	1244	4028	1592	6864
Unadjusted BSI rate per 1000 admissions	**1.549**	**7.300**^**c**^	**2.319**^**c**^	**1.032**^**c**^	**1.989**^**c**^
Age: avg ± SD, (IQR; median)	62.0 ± 16.1 (53–74; 64)	63.4 ± 13.7^a^ (54–73; 65)	61.9 ± 16.1 (52–73; 64)	62.4 ± 15.5 (53–74; 64)	62.3 ± 15.6 (53–73; 64)
Males (%)	59.2% (1640)	62.0% (771)	60.7% (2444)	59.7% (950)	60.7% (4165)
Prior 30-day admission (%)	17.5% (484)	9.8% (122)^c^	18.3% (736)	18.2% (289)	16.7% (1147)
Prior 90-day admission (%)	26.7% (741)	14.1% (175)^c^	31.2% (1255)^c^	29.0% (462)	27.6% (1892)
> 1 Underlying Conditions^d^ (%)	91.5% (2535)	98.7% (1228)^c^	94.0% (3788)^c^	90.8% (1446)	94.1% (6462)^c^
Time to HO Culture Collection: Avg ± SD (IQR; median)	11.0 ± 13.1 (3–14; 7)	17.5 ± 15.2^c^ (8–22; 14)	11.4 ± 15.4 (3–14; 7)	11.4 ± 13.7 (3–14; 7)	12.5 ± 15.1 (4–16; 8)
LOS (days): avg ± SD (IQR; median)	25.5 ± 20.1 (12–32; 20)	34.8 ± 25.2^c^ (18–44; 28)	26.7 ± 23.9^a^ (12–34; 20)	25.7 ± 24.5 (11–30; 19)	28.0 ± 24.5^c^ (13–35; 21)
% Ventilated^e^ (%)	21.5% (595)	52.9% (658)^c^	23.5% (947)^a^	25.3% (403)^b^	29.3% (2008)^c^
ICU admission (%)	55.4% (1534)	72.9% (907)^c^	53.8% (2169)	51.6% (821)^a^	56.8% (3897)
Overall LOS (days): avg ± SD (IQR; median)	28.9 ± 22.0 (14–36; 23)	37.1 ± 26.0^c^ (20–46; 30)	30.9 ± 26.1^a^ (15–38; 24)	30.4 ± 27.2 (14–36; 23)	32.3 ± 26.4^c^ (16–40; 25)
ICU LOS (days): avg ± SD (IQR; median)	12.3 ± 13.6 (3–16; 8)	23.5 ± 18.3^c^ (10.5–32; 20)	13.6 ± 16.3 (3–18; 9)	14.8 ± 15.9^a^ (4–20; 10)	16.1 ± 17.2^c^ (4–22; 11)
Hospital mortality (%)	NA	48.6% (377/775)^c^	19.7% (486/2469)	NA	26.7% (869/3252)

^a^*P* < .05; ^b^*P* < .01; ^c^*P* < .001 bivariate correlations compared to the pre-SARS-CoV-2 period

^d^Includes the following baseline conditions occurring within the first 3 days of hospital admissions as determined by the maximum value of surrogate laboratory results: renal insufficiency (serum creatinine [SCr] > 2.0 mg/dL [to convert to micromoles per liter, multiply by 88.4]); kidney failure (blood urea nitrogen > 100 and SCr > 3.0 mg/dL); suspected sepsis (lactic acid > 2.0 or > 4.0 mmol/L); suspected heart failure (brain-type natriuretic peptide [BNP] > 400 pg/mL [to convert to nanograms per liter, multiply by 1.0] or N-terminal pro BNP > 900 pg/mL); myocardial inflammation (doubling of troponin drawn within 6 h final value: troponin 0.4 ng/L [to convert to micrograms per liter, multiply by 1.0], troponin T15 ng/L [to convert to micrograms per liter, multiply by 1.0] in male patients, troponin T10 ng/L in female patients, troponin I > 0.04 ng/mL [to convert to micrograms per liter, multiply by 1.0]); liver dysfunction (any of the following: alanine aminotransferase > 60 U/L [to convert to microkatals per liter, multiply by 0.0167], aspartate aminotransferase > 80 U/L [to convert to microkatals per liter, multiply by 0.0167], serum albumin < 3.0 g/dL [to convert to grams per liter, multiply by 10], international normalized ratio > 2.0 [and not currently receiving warfarin, rivaroxaban, apixaban, edoxaban, or betrixaban]); cytokine stimulation (any of the following: fibrinogen < 250 mg/dL [to convert to grams per liter, multiply by 0.01], C-reactive protein > 7.0 mg/dL [to convert to milligrams per liter, multiply by 10], D-dimer [dimerized plasmin fragment D] > 1000 ng/mL [to convert to nanomoles per liter, multiply by 5.476], erythrocyte sedimentation rate > 30 mm/h, or triglycerides > 265 mg/dL [to convert to millimoles per liter, multiply by 0.0113])

^e^The patient was (a) started on intravenous (IV)/IV push (IVP) sedation medications (propofol, lorazepam, midazolam, dexmedetomidine, or ketamine) or IV/IVP opioids (fentanyl, remifentanil, sufentanil, or hydromorphone) with a duration ≥ 24 h and (b) ≥ 2 arterial blood gas results collected ≥ 24 h apart (on the first day of sedation medication and a subsequent result 24 h later)

Compared to patients during the pre-pandemic period, SARS-CoV-2-positive patients with a HOBSI infection were significantly older (63.4 y vs 62.0 y, *P* < 0.05) and less likely to have had a prior 30- or 90-day admission, more likely to have ≥ 1 underlying condition (98.7% vs 91.5%; *P* < 0.001), and had a longer time from admission to hospital (HO) blood culture collection (17.5 vs 11.0 days; *P* < 0.001).

During the pandemic period, the rate of HOBSI was significantly higher among SARS-CoV-2-positive patients (7.3 per 1000 admissions) and SARS-CoV-2-negative patients (2.3 per 1000 admissions) compared to the rate during the pre-pandemic period (1.5 per 1000 admissions; *P* < 0.001).

As seen with COBSI, significantly higher rates of HOBSI per 1000 admissions attributable to *S aureus* (0.58 vs. 0.45), *Enterococcus* spp (0.25 vs. 0.16), *K pneumoniae* (0.17 v. 0.14), non-*C albicans* (0.15 vs. 0.10), *C albicans* (0.13 vs. 0.08), and *P aeruginosa* (0.11 vs. 0.08), all *P* ≤ 0.0093, were observed during the pandemic compared to the pre-pandemic period.

Although the median time from admission to collection of HOBSI events was significantly longer among SARS-CoV-2-positive patients during the pandemic (14 days, *P* < 0.001) compared to the pre-pandemic period (7 days), the median time to HO culture collection was similar among SARS-CoV-2–negative patients (7 days) and among patients not tested (7 days) (Table 5).

**Table 5:** Time to collection: HO BSI positive admissions by pathogen stain, SARS-CoV-2 period and testing status

Hospital day of culture collection	Pre-SARS-CoV-2: July 2019–February 2020 (1,789,449 Admissions)					Post-SARS-CoV-2 March 2020—September 4, 2021														
						SARS-CoV-2 + (170,402 Admissions)					SARS-CoV-2—(1,737,037 Admissions)					Not Tested (1,542,804 Admissions)				
	Total	GN	GP	Fungus/ Yeast	Poly	Total	GN	GP	Fungus/ Yeast	Poly	Total	GN	GP	Fungus/ Yeast	Poly	Total	GN	GP	Fungus/ Yeast	Poly
Day 3	16.1%	14.2%	20.8%	8.4%	10.6%	3.1%	3.4%	4.1%	0.8%	0.0%	15.8%	14.6%	19.8%	8.5%	10.5%	15.5%	14.9%	18.4%	4.8%	18.2%
Day 4	26.8%	24.0%	33.8%	14.4%	23.1%	7.1%	6.3%	9.4%	3.6%	3.8%	27.7%	25.4%	33.9%	17.3%	19.6%	26.7%	26.8%	30.0%	11.8%	31.2%
Day 5	35.5%	32.1%	44.1%	18.9%	33.7%	10.2%	8.2%	14.0%	5.6%	5.8%	36.2%	32.6%	43.8%	24.7%	28.0%	34.2%	32.3%	40.0%	15.6%	40.3%
Day 6	43.1%	39.2%	52.2%	25.7%	42.3%	14.0%	10.8%	19.0%	7.6%	13.5%	43.4%	38.2%	52.5%	30.7%	39.9%	40.5%	37.2%	48.1%	20.4%	44.2%
Day 7	49.5%	46.4%	58.5%	31.1%	45.2%	17.8%	13.4%	24.2%	11.2%	13.5%	49.4%	44.8%	58.7%	35.1%	44.8%	46.9%	45.3%	54.0%	24.7%	48.1%
Day 8	54.1%	51.7%	62.8%	34.7%	49.0%	22.5%	19.2%	29.0%	14.1%	17.3%	54.9%	50.4%	64.5%	39.1%	51.0%	53.1%	51.3%	60.3%	31.2%	53.2%
Day 9	58.9%	56.5%	67.2%	40.7%	52.9%	27.4%	23.4%	34.5%	19.3%	19.2%	58.9%	54.7%	68.4%	41.8%	57.3%	57.0%	55.0%	64.8%	34.4%	55.8%
Day 10	63.0%	61.0%	70.3%	46.1%	59.6%	32.3%	29.2%	38.5%	24.9%	23.1%	62.8%	59.1%	71.2%	46.7%	63.6%	62.4%	60.6%	70.1%	40.3%	58.4%
Day 11	66.7%	65.2%	73.7%	49.7%	62.5%	36.7%	33.4%	43.5%	28.1%	26.9%	66.2%	63.0%	74.4%	50.4%	65.0%	66.5%	66.1%	73.4%	43.5%	62.3%
Day 12	69.1%	67.8%	75.7%	53.0%	63.5%	40.6%	36.6%	47.1%	34.5%	28.8%	69.5%	66.6%	77.4%	54.0%	66.4%	69.4%	70.1%	75.5%	45.7%	64.9%
Day 13	71.9%	70.7%	78.2%	56.6%	65.4%	44.9%	39.7%	52.2%	38.6%	34.6%	71.8%	68.9%	79.2%	57.1%	69.9%	72.4%	73.2%	77.4%	51.6%	68.8%
Day 14	74.7%	73.9%	80.3%	58.7%	73.1%	48.6%	42.4%	55.4%	44.2%	40.4%	74.3%	72.1%	81.1%	60.0%	72.0%	74.9%	75.3%	79.5%	57.5%	70.1%
Day 15	77.0%	76.6%	82.1%	61.7%	76.0%	52.4%	45.8%	59.5%	48.6%	42.3%	76.7%	74.7%	83.0%	63.5%	74.8%	77.8%	78.1%	81.9%	61.8%	75.3%
> Day 15	100.0%	100.0%	100.0%	100.0%	100.0%	100.0%	100.0%	100.0%	100.0%	100.0%	100.0%	100.0%	100.0%	100.0%	100.0%	100.0%	100.0%	100.0%	100.0%	100.0%
**Total HO Admissions**	**2771**	**1188**	**1145**	**334**	**104**	**1244**	**380**	**563**	**249**	**52**	**4028**	**1610**	**1725**	**550**	**143**	**1592**	616	713	186	77
HO BSI/1000 Adm	1.55	0.66	0.64	0.19	0.06	7.30^b^	2.23^b^	3.30^b^	1.46^b^	0.31^b^	2.32^b^	0.93^b^	0.99^b^	0.32^b^	0.08^b^	1.03^b^	0.40^b^	0.46^b^	0.12^b^	0.05
Time to HO culture collection																				
Avg ± SD (Median)	11.0 ± 13.1 (7)	11.2 ± 12.6 (7)	9.5 ± 12.4 (5)	15.8 ± 16.8 (11)	10.5 ± 8.9 (8)	17.5 ± 15.2^b^ (14)^b^	19.8 ± 16.1^b^ (16)	15.8 ± 15.2^b^ (12)	17.4 ± 11.8 (15)	20.9 ± 18.6^b^ (16.5)	11.4 ± 15.4 (7)	12.4 ± 18.1^a^ (7)	9.6 ± 13.4 (5)	13.8 ± 12.3 (10)	11.3 ± 11.5 (7)	11.4 ± 13.7 (7)	11.3 ± 12.9 (7)	9.5 ± 10.4 (6)	17.9 ± 12.8 (12)	12.6 ± 16.4 (7)

^a^*P* < 0.05 for bivariate correlations compared to the pre-SARS-CoV-2 period; ^b^*P* ≤ 0.00662 for bivariate correlations compared to the pre-SARS-CoV-2 period

### Outcomes

Overall, outcomes among patients diagnosed with BSI in the pre-pandemic period differed from those during pandemic period (Tables 2 and 4). These differences were largely driven by differences between SARS-CoV-2-positive patients and those who were negative or not tested. Among patients with COBSI, SARS-CoV-2-positive patients had significantly higher rates of ICU admissions (36.6% vs 32.8%; *P* < 0.01) and mechanical ventilation (10.7% vs 4.7%; *P* < 0.01), with significantly longer overall and ICU LOS (overall LOS: 12.2 vs 9.1 days; *P* < 0.001; ICU LOS; 6.5 vs 4.5 days; *P* < 0.001) compared to patients in the pre-pandemic period. Among the subset of patients during the pandemic period for whom mortality data were available (n = 20,064, 50.5%), mortality was 20.7% among SARS-CoV-2-positive patients and 7.0% among SARS-CoV-2-negative patients.

Among patients with HOBSI, rates of ICU admission were similar between the pre-pandemic and pandemic periods; however, during the pandemic period, rates of ventilator use (29.3% vs 21.5%; *P* < 0.001), hospital LOS (28.0 vs 25.5 days; *P* < 0.001) and ICU LOS were higher (16.1 vs 12.3 days; *P* < 0.001). These differences were likely driven by SARS-CoV-2-positive patients, who, compared with patients during the pre-pandemic period, had higher rates of ICU admission (72.9% vs 55.4%; *P* < 0.001), ventilator use (52.9% vs 21.5%; *P* < 0.001), and longer hospital LOS and ICU LOS (hospital LOS: 34.8 vs 25.5 days; *P* < 0.001; ICU LOS, 23.5 vs 12.3; *P* < 0.001). Among the subset of patients with HOBSI during the pandemic for whom mortality data were available (n = 3252), mortality rates were significantly higher among SARS-CoV-2-positive patients (48.6%) compared to SARS-CoV-2–negative patients (19.7%; *P* < 0.001) (Table 4).

## Discussion

This study showed a significantly higher blood culture positive rate for both COBSI and HOBSI in the pandemic period compared to the pre-pandemic period. The higher rate of COBSIs during the pandemic was primarily driven by higher rates in SARS-CoV-2-negative patients whereas the higher rates of HOBSIs were primarily driven by rates in SARS-CoV-2-positive patients. These findings are consistent with those from a recent, large meta-analysis of 46 studies (published through April 19, 2021) including 42,694 patients [15], which reported that 7.3% of patients hospitalized with COVID-19 developed a BSI (95% CI, 4.7–1.1%) and that BSIs were nearly 3 times more likely in COVID-19-positive patients compared to those without COVID-19 (OR 2.77; 95% CI, 1.53–5.02; *P* < 0.001) [15].

Our study is among the first reports to compare BSI rates and causative pathogens during the pandemic with those observed prior to the pandemic. A particularly interesting finding from our analysis is the substantially higher rates of COBSI among SARS-CoV-2-negative patients (15.8 per 1000 admissions) compared with SARS-CoV-2-positive patients (9.6 per 1000 admissions).

Higher HOBSI rates during the pandemic may have reflected high levels of patient severity and long ICU and hospital lengths of stay. It is possible that intrinsic factors may also have adversely impacted outcomes in patients without SARS-CoV-2, resulting in higher HOBSI rates. For example, overworked hospital staff, widespread during the pandemic period [16] could have led to suboptimal care, contributing to increased HOBSI rates. Short staffing has been associated with a higher incidence of HO infections and data from the CDC’s National Safety Health Network indicate that the higher rates of several important HO infections in 2020 versus 2019 [17]. In fact, the CDC analysis demonstrated that the rate of central-line associated bloodstream infections (CLABIs) were nearly 50% higher in the latter half of 2020 and methicillin-resistant *Staphylococcus aureus* (MRSA) infections increased by 34% compared with 2019 [17]. Moreover, because prior studies have shown that central line-associated infection is a common cause of BSIs in patients with COVID-19, a fear of prolonged patient contact, the practice of pronation for ventilated patients [18], and aerosolization of SARS-CoV-2 could be a barrier to catheter hygiene and maintenance [7]. Finally, the pandemic has revealed staffing constraints and supply chain weaknesses that limit the ability of microbiology labs and infectious disease specialists to fully support infection prevention and antimicrobial stewardship policies, which also may affect BSI rates and outcomes [19, 20].

The increased rate of COBSI admissions could also be attributable to delays in care [21]. A survey of 5,412 US adults revealed that approximately 41% delayed or avoided medical care during the pandemic due to concerns about COVID-19, including 12% who avoided urgent or emergency care and 32% who avoided routine care. Results from a retrospective analysis of Medicare beneficiaries reported early increases in hospitalizations and 30-day mortality, suggesting that patients were presenting with more advanced illness, possibly related to delays in care [22, 23].

Differences were also identified in blood-positive pathogens during the pre-pandemic and pandemic periods. For example, rates of *Enterococcus* were higher during the pandemic, a finding that confirms the previous results that have shown an unexpectedly high prevalence of enterococcal BSIs among patients with COVID-19 [24–28]. Compared to the pre-pandemic period, we also found higher rates of HO fungal BSI caused by *Candida* species during the pandemic, confirming data suggesting high fungal BSI rates among SARS-CoV-2-positive patients [7, 29]. In our study, *C albicans* was the third most common pathogen and seen in 10.5% of SARS-CoV-2-positive patients but were not observed as a top 5 pathogen among SARS-CoV-2-negative patients or in the pre-pandemic period (Table 3). A significant 35% increase in HOBSI risk associated with *Candida* species during the pandemic period has also been observed in a study of 69 US hospitals [30], possibly driven by risk factors frequently present in critically ill patients with COVID-19, including the use of mechanical ventilation, indwelling devices, glucocorticoids, and broad-spectrum antibiotic therapy [31–33]. Our results also showed a significantly longer time for culture collection in HOBSI pathogens in SARS-CoV-2-positive patients (14 days median) compared to SARS-CoV-2-negative patients, patients not tested, and the pre-pandemic period (median 7 days for all 3 groups).

Key strengths of our study include its demographic and geographic diversity, and its duration, including data from multiple phases within the first 18 months of the pandemic. These strengths address the limitations of previous studies, which have generally been smaller, less geographically or demographically representative of the US population, and of a relatively short duration early in the pandemic [6, 25, 28, 34, 35]. Lastly, our study included a comparison with pre-COVID-19 patients to examine potential differences between the pre- and post-pandemic period. Accordingly, we found significantly higher hospital-onset BSI rates/1000 admissions during the pandemic period compared to the pre-pandemic period for several pathogens, including *S aureus*, *Enterococcus* spp*, K pneumoniae*, non-*C albicans*, *C albicans*, and *P aeruginosa*. Further evaluation of prevalence of these pathogens in SARS-2-CoV-associated BSI is warranted, particularly for *Candida* and *Enterococcus*.

Our study also had several limitations. First, we did not evaluate the impact of medications and immunosuppressive therapies on hospital-onset BSI rates. A multicenter study of hospitalized adults with COVID-19 found that the combined use of corticosteroids and tocilizumab in these patients was associated with BSI rates, independent of other risk factors [36]. However, results from a small single-center study suggested that nosocomial use of dexamethasone had no effect on BSI rates in the critically ill [10]. Further studies are needed to establish any definitive pathophysiologic role for immunosuppressive therapy in the development of BSIs in patients with COVID-19. We were also unable to evaluate or document markers of hospital stressors, including staffing limitations, lack of ICU space, and shortages in personal protective equipment, which might affect BSI rates or patient outcomes. Finally, we cannot draw conclusions regarding the impact of the pandemic on BSI-related mortality because pre-pandemic mortality data were not available.

In conclusion, overall rates of BSIs have appeared to significantly increase during the COVID-19 pandemic, largely resulting from an increase in COBSI rates among SARS-CoV-2-negative patients and HOBSI rates among both SARS-CoV-2- positive and negative patients. The additive burden of BSI and COVID-19 highlights a need for interventions to both prevent and effectively treat hospital-acquired BSI in SARS-CoV-2-positive patients. Identification and evaluation of such interventions during later stages of the pandemic is warranted.

## Supplementary Information

**Additional file 1: Table S1.** Distribution of study hospitals.

**Additional file 2: Table S2.** Laboratory criteria used as surrogates for admission-period clinical conditions.

## Data Availability

The datasets used and/or analyzed in the current study are available from the corresponding author on reasonable request. The data sharing policy of Merck Sharp and Dohme Corp, a subsidiary of Merck and Co, Inc, including restrictions, is available at http://engagezone.msd.com/ds_documentation.php. Requests for access to the study data can be submitted through the EngageZone site or via email to moc.kcrem@sseccaatad.
