# Supplementary material for: Epidemiology and outcomes of culture-positive bloodstream pathogens prior to and during the SARS-CoV-2 pandemic: a multicenter evaluation
Source: BMC Infect Dis. 2022 Nov 11;22:841. doi: 10.1186/s12879-022-07810-8 (PMC9651895; doi:10.1186/s12879-022-07810-8)
Supplement: Supplementary file 1 — Additional file 1: Table S1. Distribution of study hospitals. [file 12879_2022_7810_MOESM1_ESM.docx]

**Table-S1. Distribution of Study Hospitals**

| **Characteristics** | **#Hospitals** | |  |  |  |  |
| --- | --- | --- | --- | --- | --- | --- |
|  | **n** | **%** |  |  |  |  |
| **Overall** | 271 | 100.0 |  |  |  |  |
| **Year**^a^ |  |  |  |  |  |  |
| Pre-SARS-CoV-2 | 267 | 98.5 |  |  |  |  |
| Post-SARS-CoV-2 | 271 | 100.0 |  |  |  |  |
| **Bed size** |  |  |  |  |  |  |
| Less than 100 | 96 | 35.4 |  |  |  |  |
| 100-300 | 108 | 39.9 |  |  |  |  |
| Greater than 300 | 67 | 24.7 |  |  |  |  |
| **Urban/Rural** |  |  |  |  |  |  |
| Rural | 94 | 34.7 |  |  |  |  |
| Urban | 177 | 65.3 |  |  |  |  |
| **Teaching status** |  |  |  |  |  |  |
| Non-teaching | 181 | 66.8 |  |  |  |  |
| Teaching | 90 | 33.2 |  |  |  |  |
| **Census region**^b^ |  |  |  |  |  |  |
| Region 1 (East North Central) | 42 | 15.5 |  |  |  |  |
| Region 2 (East South Central) | 36 | 13.3 |  |  |  |  |
| Region 3 (Middle Atlantic) | 38 | 14.0 |  |  |  |  |
| Region 4 (Mountain) | 11 | 4.1 |  |  |  |  |
| Region 5 (New England) | 5 | 1.8 |  |  |  |  |
| Region 6 (Pacific) | 27 | 10.0 |  |  |  |  |
| Region 7 (South Atlantic) | 41 | 15.1 |  |  |  |  |
| Region 8 (West North Central) | 16 | 5.9 |  |  |  |  |
| Region 9 (West South Central) | 55 | 20.3 |  |  |  |  |
| ^a^ The numbers of hospitals in the pre- and post- SARS-CoV-2 period do not add up to 271 as hospital count changes year to year. | | | | | | |
| ^b^ Census regions are defined in the below (only states in data sample included). | | | | | | |
| Region 1 (East North Central): Illinois, Indiana, Michigan, Ohio, and Wisconsin. | | | | |  |  |
| Region 2 (East South Central): Alabama, Kentucky, Mississippi, and Tennessee. | | | | | | |
| Region 3 (Middle Atlantic): New Jersey, New York, and Pennsylvania. | | | | |  |  |
| Region 4 (Mountain): Arizona, Idaho, Montana, and New Mexico | | | | |  |  |
| Region 5 (New England): Connecticut and New Hampshire. | | | | |  |  |
| Region 6 (Pacific): California, Oregon, and Washington. | | | | |  |  |
| Region 7 (South Atlantic): Delaware, Georgia, Florida, Maryland, North Carolina, South Carolina, Washington DC, West Virginia, and Virginia. | | | | |  |  |
| Region 8 (West North Central): Iowa and Missouri. | | | | |  |  |
| Region 9 (West South Central): Louisiana, Oklahoma, and Texas | | | | |  |  |
