# Supplementary material for: Epidemiology and outcomes of culture-positive bloodstream pathogens prior to and during the SARS-CoV-2 pandemic: a multicenter evaluation
Source: BMC Infect Dis. 2022 Nov 11;22:841. doi: 10.1186/s12879-022-07810-8 (PMC9651895; doi:10.1186/s12879-022-07810-8)
Supplement: Supplementary file 2 — Additional file 2: Table S2. Laboratory criteria used as surrogates for admission-period clinical conditions. [file 12879_2022_7810_MOESM2_ESM.docx]

**Table-S2. Laboratory Criteria Used as Surrogates for Admission-period Clinical Conditions.**Values recorded within the first 3 days following admission were considered to indicate the existence of the designated condition.

| **Clinical condition** | **Laboratory criteria** |
| --- | --- |
| Renal insufficiency/failure | Serum creatinine > 2.0 mg/dL |
| Renal failure | Blood urea nitrogen >100 mg/dL AND serum creatinine > 3.0 mg/dL |
| Sepsis | Serum lactate >2.0 mmol/L (sepsis) or >4.0 mmol/L (severe sepsis) |
| Suspected heart failure | Brain natriuretic peptide (BNP) > 400 pg/mL OR N-terminal BNP  > 900 pg/mL |
| Myocardial inflammation | 2-fold elevation in troponin drawn within 6 hours and a final value of troponin ≥ 0.4 ng/L, troponin T ≥ 15 ng/L in males and ≥ 10 ng/L in females, or troponin I > 0.04 ng/mL |
| Liver dysfunction | Any of the following: alanine aminotransferase > 60 U/L, aspartate aminotransferase > 80 U/L, serum albumin < 3.0 gm/dL, international normalized ratio >2.0 [and not currently receiving warfarin, rivaroxaban, apixaban, edoxaban, or betrixaban] |
| Cytokine stimulation | Any of the following: fibrinogen < 250 mg/dL, C-reactive protein > 70 mg/L, D-dimer >1000 ng/mL, erythrocyte sedimentation rate > 30 mm/hr, triglycerides > 265 mg/dL |
| Diabetes | Non-insulin diabetes medications prescribed during hospitalization or hemoglobin A1c > 6.5% in the last 6 months |
